# Synthesis of ppGpp impacts type IX secretion and biofilm matrix formation in *Porphyromonas gingivalis*

**DOI:** 10.1038/s41522-020-0115-4

**Published:** 2020-01-31

**Authors:** Hey-Min Kim, Mary E. Davey

**Affiliations:** Department of Oral Biology, College of Dentistry, University of Florida, Gainesville, FL USA

**Keywords:** Biofilms, Bacteriology

## Abstract

In order to persist, bacteria need to adjust their physiological state in response to external and internal cues. External stimuli are often referred to as stressors. The stringent response, mediated by the alarmone (p)ppGpp, is central to the stress response in many bacteria; yet, there is limited knowledge regarding the role of (p)ppGpp signaling in bacteria belonging to the phylum Bacteroidetes. Like its counterparts in the gut (e.g., *Bacteroides thetaiotaomicron* and *Bacteroides fragilis*), *Porphyromonas gingivalis* persists in close association with its human host. Given the potential for numerous perturbations in the oral cavity, and the fact that *P. gingivalis* can enter and replicate within host cells, we hypothesized that (p)ppGpp is a key signaling molecule for stress adaptation and persistence. Here, we show that accumulation of ppGpp in *P. gingivalis* is governed by two homologous enzymes, designated Rel, and RshB, and that ppGpp signaling affects growth rate, survival, biofilm formation, production of outer membrane vesicles, and expression of genes encoding type IX secretion structural and cargo proteins. Overall, our findings provide a potential mechanism by which biofilm formation and virulence of *P. gingivalis* are integrated via ppGpp signaling, a regulatory mechanism central to bacterial survival in dynamic environments.

## Introduction

*Porphyromonas gingivalis* is an anaerobic bacterium belonging to the phylum Bacteroidetes that persists within the oral microbiome, and is strongly associated with the progression of chronic periodontitis.^1,2^ This bacterium is metabolically unusual, it obtains its iron from heme,^3,4^ and it does not metabolize sugars; instead, it is highly proteolytic, obtaining its carbon, nitrogen, and energy from protein substrates.^5–8^ For this reason, secretion of proteolytic enzymes into its environment is central to *P. gingivalis* physiology. The type IX secretion system (T9SS), which is restricted to the phylum Bacteroidetes, is a complex translocon that accomplishes the secretion of over 30 *P. gingivalis* proteins bearing a specific C-terminal domain, including the trypsin-like gingipains, which are key virulence determinants.^9^ Another key secretion mechanism exploited by *P. gingivalis* is production of outer membrane vesicles (OMVs). *P. gingivalis* is highly proficient in OMV production, and not surprisingly, these secreted vesicles are decorated with T9SS cargo proteins, thus providing an effective means to promote the spread of proteolytic enzymes into the surroundings for nutrient acquisition, as well as the spread of virulence determinants.^10,11^ Although what initiates chronic periodontal disease is still a key question, it is broadly accepted that inflammation and an increased flow of gingival crevicular fluid results in a shift in microbiota to primarily Gram-negative anaerobes, which includes *P. gingivalis*.^12^ Recent reports indicate that periodontitis is also strongly linked to systemic inflammatory disorders such as diabetes, Alzheimer’s disease, cardiovascular disease, and rheumatoid arthritis.^13–17^ In this context, it is important to increase our basic understanding of *P. gingivalis* physiology in regard to the mechanisms that control its survival and virulence.

Bacteria that persist within the human oral microbiome encounter diverse stressors including pH shifts, oxidative stress, and nutrient limitation, and they adjust their physiology for survival.^18,19^ The intracellular alarmones, guanosine 5′-diphosphate 3′-diphosphate (ppGpp) and guanosine 5′-triphosphate 3′-diphosphate (pppGpp), are key signaling molecules that enable cell homeostasis.^20^ It has been reported that changes in the intracellular level of (p)ppGpp can be triggered by various environmental perturbations, including changes in nutrient availability,^21^ redox,^22^ pH,^23^ and temperature.^24^ The cellular stress response mediated by the increased level of (p)ppGpp is commonly referred to as the stringent response.^25^ Initial studies on the stringent response focused on the Gram-negative model organism *Escherichia coli*, which has two multi-domain enzymes: RelA and SpoT.^26^ RelA-SpoT homologs (RSHs) in other bacteria are key enzymes that synthesize and degrade (p)ppGpp.^20,25^ The synthase domain (SD) of RSH enzymes catalyzes the transfer of pyrophosphate from ATP to either GDP or GTP to generate ppGpp or pppGpp, respectively, while the RSH hydrolase domain (HD) degrades (p)ppGpp to pyrophosphate and either GDP or GTP. In general, (p)ppGpp levels affect bacterial transcription, translation, and DNA replication, but the mechanisms by which these second messenger nucleotides regulate these cellular processes vary greatly.^26–28^ Studies have also shown that (p)ppGpp signaling is important for diverse biological processes such as antibiotic resistance,^29^ biofilm formation,^30^ colonization,^31^ persistence,^32^ survival during host invasion,^33^ and virulence.^34,35^ Importantly, multiple pathogens use (p)ppGpp signaling to mediate production of virulence factors. The multiplicity of the bacterial genes and regulatory pathways influenced by the (p)ppGpp signaling suggests that the relationship between (p)ppGpp signaling and virulence could be unique for each pathogen. To date, there is limited knowledge regarding the role of (p)ppGpp signaling in bacteria belonging to the phylum Bacteroidetes; in particular, the role of (p)ppGpp signaling in *P. gingivalis* stress adaptation or virulence has not been investigated. In this study, we generated three deletion mutants in strain 381 in genes predicted to be involved in regulating the intracellular concentrations of ppGpp (*Δrel*, *ΔrshB*, and *ΔrelΔrshB*), and investigated the ppGpp levels and phenotypes in these strains. We compared growth, viability, biofilm formation, morphology, virulence, and the transcriptome between parent strain 381 and its derivatives, and we evaluated the effect of heme deprivation on ppGpp levels. Overall, our data indicate that ppGpp directly or indirectly affects stress adaptation, biofilm matrix composition, production of outer membrane vesicles, and the pathogenic potential of *P. gingivalis*.

## Results

### *P. gingivalis* harbors two enzymes that regulate ppGpp levels whose function is impacted by the availability of hemin

The genome of *P. gingivalis* strain 381 harbors two genes predicted to encode multi-domain RSH enzymes,^18^ PGN_0465 (PGF_RS02215 in strain 381) and PGN_1757 (PGF_RS08445 in strain 381). As expected, the genes share greater sequence similarity with BT0700 and BT3998 from *Bacteroides thetaiotaomicron*, respectively, than they do either to RelA or SpoT from *Escherichia coli* (Supplementary Fig. 1).^31^ The data suggest that an RSH gene duplication occurred in bacteria belonging to the phylum Bacteroidetes. Because the nomenclature Rel and RshB for the ancestral bifunctional RSH has been described in the genera *Porphyromonas* and *Bacteroides*,^20^ hereafter we refer to the PGN_0465 and PGN_1757 as Rel and RshB, respectively.

**Fig. 1:**
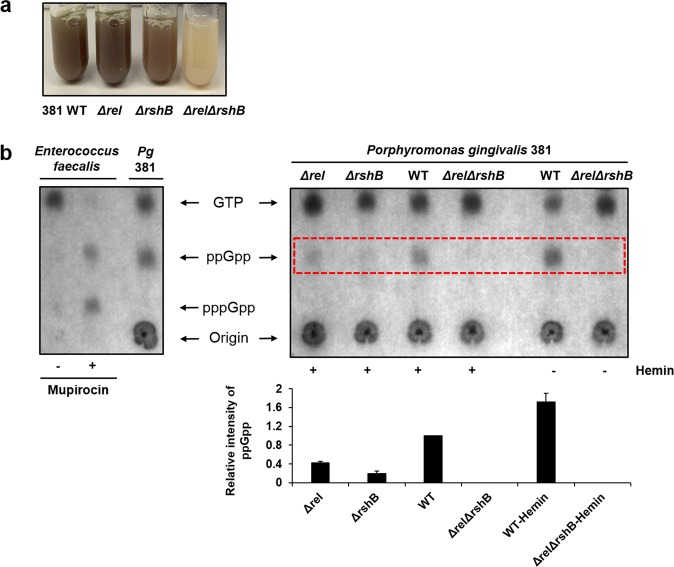
*P. gingivalis* encodes two RelA–SpoT homolog (RSH) proteins that contribute to ppGpp accumulation, and hemin starvation can activate the ppGpp accumulation. **a** When grown overnight to the stationary phase in TSBHK, the ppGpp^0^ mutant (*ΔrelΔrshB*) lacks pigmentation, indicative of a defect in heme binding. **b** Thin-layer chromatography of ^32^P-labeled extracts from *Enterococcus faecalis* (control) or *P. gingivalis* cells in the absence and presence of mupirocin or hemin shows that the *ΔrelΔrshB* does not synthesize ppGpp, and that the wild type (WT) accumulates ppGpp in response to hemin limitation. Quantification of ppGpp levels was performed by using ImageJ software. The data provided in the lower graph show the average of two biological replicates; error bars depict standard deviation.

In this study, we generated *Δrel*, *ΔrshB*, and *ΔrelΔrshB* mutants in *P. gingivalis* strain 381. Interestingly, as shown in Fig. 1a, while no phenotype was observed in the single mutants, the double mutant (*ΔrelΔrshB*) exhibited less heme-binding capacity (black pigmentation), especially in broth culture. Since heme plays an essential role in the growth, protection against oxidative stress, and virulence of *P. gingivalis*,^3,36^ we hypothesized that hemin deprivation could be a key stressor that triggers accumulation of (p)ppGpp in *P. gingivalis*. To determine whether Rel and RshB are indeed required for (p)ppGpp synthesis, and if hemin has an effect on *P. gingivalis* (p)ppGpp accumulation, we analyzed ^32^P-labeled nucleotide extracts by thin-layer chromatography in the presence and absence of 1 µg ml^−1^ hemin. Since ppGpp and pppGpp accumulate in *Enterococcus faecalis* in the presence of mupirocin,^37^ cells of *E. faecalis* were used as a positive control of ppGpp and pppGpp. As shown in Fig. 1b, *P. gingivalis* produced ppGpp, which migrates between GTP and the origin; however, unlike *E. faecalis*, *P. gingivalis* only produced ppGpp; guanosine pentaphosphate (pppGpp) was not detected. Quantification of ppGpp levels revealed that the parent strain accumulates 2.36 times more ppGpp than the *rel* mutant and 5.16 times more ppGpp than the *rshB* mutant. The ppGpp profiles of *Δrel* and *ΔrshB* mutants revealed that RshB is the major enzyme responsible for the accumulation of ppGpp, while Rel appears to be responsible for maintaining basal levels under these growth conditions. The nucleotide ppGpp was not detected in the *ΔrelΔrshB* mutant. Based on these results, we designated the *ΔrelΔrshB* mutant as ppGpp^0^.

Previous attempts by other labs to generate a *P. gingivalis ΔrshB* mutant were unsuccessful,^18,38^ and a recent study in *B. thetaiotaomicron* by Schofield et al. reported that *rshB* gene (*bt3998*) was also recalcitrant to transposon insertion, suggesting that *rshB*-dependent (p)ppGpp hydrolase activity may be required for viability.^31^ To better understand the probable function of Rel and RshB, we generated a sequence alignment of the Rel and RshB proteins from *P. gingivalis* and *B. thetaiotaomicron* along with the (p)ppGpp synthase/hydrolase protein RelSeq from *Streptococcus equisimilis* (Supplementary Table 1). Since RelSeq has been functionally characterized,^39^ we used this information to determine if there were any changes in key residues in the hydrolysis and/or synthesis domains. As expected, the Rel and RshB proteins from *P. gingivalis* and *B. thetaiotaomicron* showed high identity; yet, the hydrolase domains in Pg_Rel and BT_0700 lacked many of the key residues. Altogether, the data indicate that Rel is a synthase (monofunctional), while RshB has both synthase and hydrolase activity. Importantly, since *rshB* was recalcitrant to transposon insertion in *B. thetaiotaomicron* and predicted to be the only RSH with hydrolase activity, we hypothesized that a mutation in *rel* could have occurred when we generated the *rshB* mutant in *P. gingivalis*. To evaluate, we cloned and sequenced *rel* and its promoter region from the *rshB* mutant, and verified that the sequence matched the parent strain. In addition, RNA-Seq analysis confirmed that the expression of *rel* was not downregulated in the *rshB* mutant. In fact, qPCR analysis (see Fig. 3 below) indicated that *rel* was slightly upregulated in the *rshB* mutant, suggesting that under our laboratory growth condition, synthesis of ppGpp by Rel in *P. gingivalis* strain 381 is low enough to not be inhibitory. Last, our data show that wild-type *P. gingivalis* increased the level of ppGpp 1.72-fold in response to hemin deprivation, but no ppGpp was detected in the *ΔrelΔrshB* mutant even under conditions of hemin deprivation (Fig. 1b). Overall, these results show that *P. gingivalis* strain 381 encodes two RSH proteins, and heme deprivation can activate ppGpp accumulation in *P. gingivalis*.

### Absence of ppGpp resulted in abnormal growth and decreased viability

**Fig. 2:**
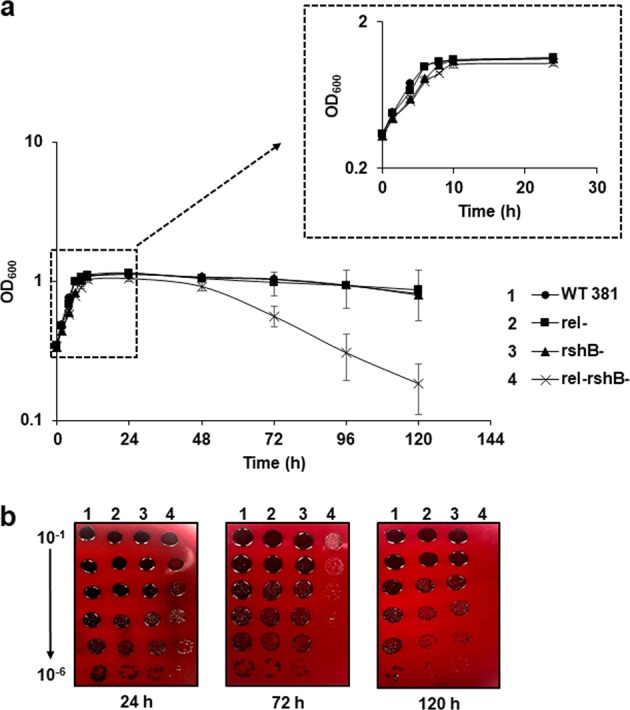
Growth curve showing that the *ΔrelΔrshB* mutant has a survival defect during the stationary phase. **a** *P. gingivalis* 381 (circles), *Δrel* (squares), *ΔrshB* (triangles), and *ΔrelΔrshB* (crosses) were grown in CDMTHK media. Points indicate the mean values, and error bars indicate standard deviations from three replications. **b** The *ΔrelΔrshB* mutant was less viable than other strains in the stationary phase. During the growth observation, cultures of the parent strain 381, *Δrel*, *ΔrshB*, and *ΔrelΔrshB* at 24, 72, and 120 h, were serially diluted, and 10 μl of each dilution from 10^−1^ to 10^−8^ was spotted on blood agar plate. The plate was removed from the anaerobic chamber and photographed after 7 days of incubation.

Recent studies have shown that ppGpp controls a wide range of biological processes such as growth rate and viability.^26,40^ Having determined that deletion of *rel* or *rshB* affects the levels of ppGpp in *P. gingivalis*, we then evaluated the impact of a lack of ppGpp synthesis and hydrolysis on bacterial growth rate and viability. As shown in Fig. 2a, the *ΔrshB* and *ΔrelΔrshB* mutants grew slightly slower than the parent and *Δrel* strains. Interestingly, in contrast to the other three strains that exhibited a gradual decline during the late stationary phase, the *ΔrelΔrshB* mutant showed a rapid decline. To verify that this rapid decline in optical density was due to lysis/cell death, we did serial dilutions of the cultures during the growth observation, and spotted 10 μl of dilutions on blood agar plates to determine viable counts (Fig. 2b). As shown in Fig. 2b, the parent strain along with *Δrel* and *ΔrshB* showed relatively stable viability from 24 to 120 h, whereas, the *ΔrelΔrshB* mutant showed a dramatic decrease in viability, as expected.

**Fig. 3:**
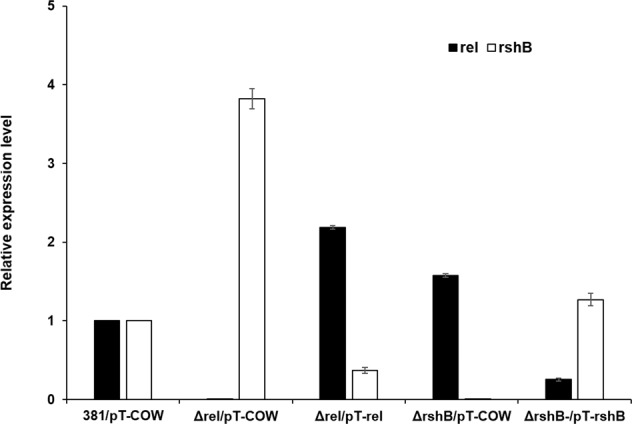
Quantitative PCR (qPCR) analysis for the complementation of the *rel* or *rshB* gene in *P. gingivalis* strain 381. *P. gingivalis* strain 381 and its derivatives were inoculated in TSBHK and grown for ~24 h to isolate RNA. The relative transcript levels of genes involved in ppGpp accumulation, including Rel (PGN_0465) and RshB (PGN_1757), were determined on the *Δrel* or *ΔrshB* deletion mutant harboring the empty plasmid (pT-COW) or pT-rel and pT-rshB. The results are presented as the relative levels (mean ± S.D. of triplicate determinations) compared with the transcript levels of the parent strain 381 harboring the empty plasmid (pT-COW). Error bars represent standard deviations of triplicate replicates.

To examine the impact of *rel* deletion (or complementation) on *rshB* transcript levels and that of *rshB* deletion (or complementation) on *rel* transcript levels, we generated plasmids expressing *rel* or *rshB* under the control of their native promoters by using plasmid pT-COW, and transformed them into the corresponding deletion mutants, and determined the expression levels of *rel* and *rshB* (qPCR). The relative transcript levels of *rel* and *rshB* were determined in the deletion mutants harboring the empty plasmid (pT-COW), pT-rel, or pT-rshB. As shown in Fig. 3, the data indicate that when *rel* is deleted, *rshB* is upregulated, and when *rshB* is deleted, expression of *rel* is upregulated, when compared with the parent strain (all containing the empty plasmid pT-COW). Further, the data show that when *rel* or *rshB* are provided *in trans* on a plasmid (pT-rel or pT-rshB) in their corresponding mutants, this inversely affects the expression levels of the other gene.

**Fig. 4:**
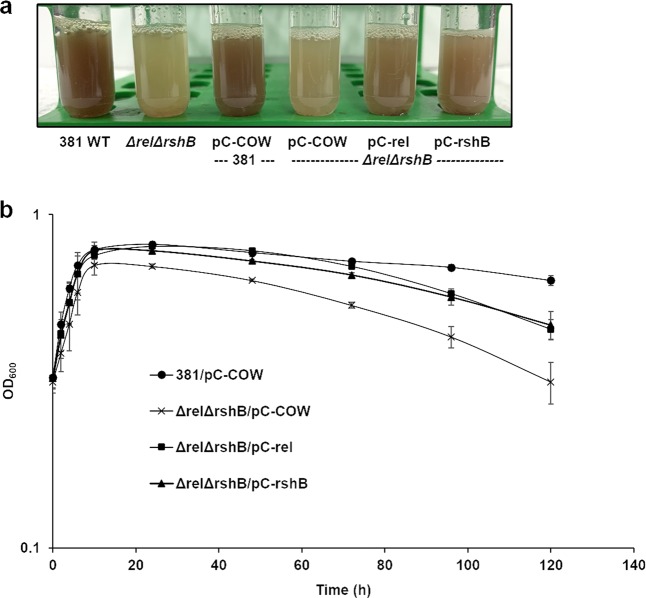
Assessment of pigmentation and growth/viability phenotypes of *P. gingivalis* 381 and *ΔrelΔrshB* containing pC-COW (empty vector controls) and complemented *ΔrelΔrshB* (pC-rel or pC-rshB). **a** When grown overnight to the stationary phase in TSBHK, the ppGpp^0^ mutant (*ΔrelΔrshB*) lacks pigmentation, indicative of a defect in heme binding. When the deletion mutant was complemented by any one of the ppGpp synthesis genes from a plasmid (pC-rel or pC-rshB), the pigmentation was partially restored. **b** *P. gingivalis* 381/pC-COW (circles), *ΔrelΔrshB*/pC-COW (crosses), *ΔrelΔrshB*/pC-rel (squares), and *ΔrelΔrshB*/pC-rshB (triangles) were grown in CDMTHK media in the presence of chloramphenicol. Points indicate the mean values, and error bars indicate standard deviations from three replications.

Since the *ΔrelΔrshB* double-deletion mutant harbors two antibiotic resistance genes (*ermF* and *tetQ*), we generated complementation plasmids that express either *rel* or *rshB* under the control of their native promoters using plasmid pC-COW that confers chloramphenicol resistance. We then complemented the *ΔrelΔrshB* mutant *in trans* (*ΔrelΔrshB*/pC-rel or *ΔrelΔrshB*/pC-rshB). We also generated control strains with the empty vector, pC-COW. Complementation of *ΔrelΔrshB* partially restored both pigmentation and the growth/viability defects (Fig. 4). Collectively, our studies demonstrate that ppGpp contributes to growth rate and survival of *P. gingivalis* during the stationary phase.

### Synthesis of ppGpp affects biofilm formation in *P. gingivalis*

**Fig. 5:**
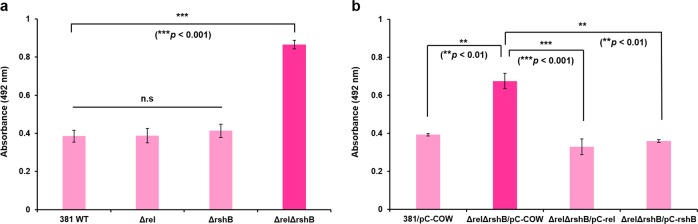
The deletion of both *rel* and *rshB* enhances biofilm formation. **a** The biomass of *P. gingivalis* 381, *Δrel*, *ΔrshB*, and *ΔrelΔrshB* after 48 h was quantified by staining with safranin. **b** The biomass of *P. gingivalis* 381/pC-COW, *ΔrelΔrshB*/pC-COW, *ΔrelΔrshB*/pC-rel, and *ΔrelΔrshB*/pC-rshB after 48 h was quantified by staining with safranin. Data are averages of three replicates (*n* = 3). Error bars represent the standard deviation. Statistical significance for the ability of biofilm formation was analyzed using the Student’s t test.

To examine the role of ppGpp in biofilm formation, we compared the biofilm-forming ability of the parent strain in comparison with the mutants in polystyrene microtiter plates. We measured biofilm biomass by safranin staining, and found that the *ΔrelΔrshB* deletion mutant had an enhanced biofilm phenotype after incubation for 48 h, with an average absorbance at 492 nm (*A*_492_) of 0.87 ± 0.02 compared with an *A*_492_ of 0.39 ± 0.03 for the parent strain (Fig. 5a). As shown in Fig. 5a, *Δrel* and *ΔrshB* mutants showed similar amount of biofilm as the parent strain. Biofilm architectures were compared between the parent strain and *ΔrelΔrshB* mutant by staining with SYTO 9 to stain all bacterial cells and propidium iodide to detect dead cells. Fluorescence microscopy confirmed that *ΔrelΔrshB* biofilms were composed of more biomass (Supplementary Fig. 2). The *ΔrelΔrshB* biofilms were also comprising more dead bacterial cells than parent strain biofilms. The presence of the pC-COW control plasmid lowered the A_492_ of *ΔrelΔrshB* to 0.68 ± 0.04 from 0.87 ± 0.02, but *ΔrelΔrshB*/pC-COW biomass was still significantly greater than that of 381/pC-COW (Fig. 5b). Importantly, complementation of *ΔrelΔrshB* restored the biofilm to wild-type levels. Taken together, these results show that the absence of ppGpp can enhance biofilm formation in *P. gingivalis* strain 381.

### The ppGpp^0^ mutant produces an altered biofilm matrix

**Fig. 6:**
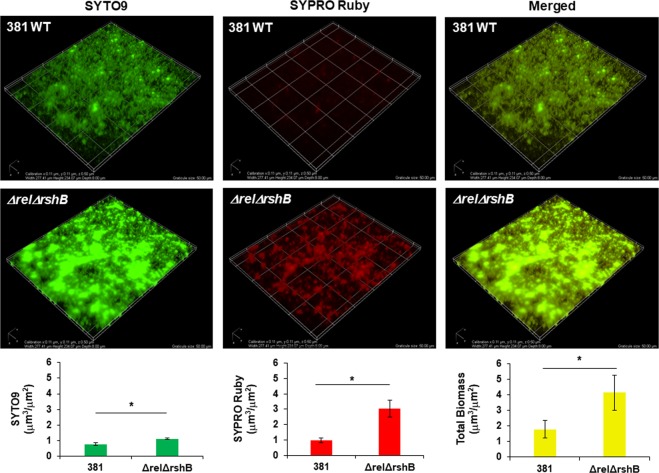
*ΔrelΔrshB* biofilms comprising more bacterial cells and protein(s) than biofilms of parent strain 381. Parent strain 381 and *ΔrelΔrshB* mutant were grown for 48 h on glass, and stained with SYTO9 (green color for cells) and SYPRO Ruby (red color for extracellular protein), and three-dimensional z stacks were acquired by fluorescence microscopy. A complete z stack (17 images per stack) was collected using Nikon imaging software (NIS-elements AR 4.30.02, 64-bit). A graticule size: 50 µm (width: 277.41 µm, height: 234.07 µm, and depth: 8 µm). The images shown are representative of three independent experiments. SYTO9, SYPRO Ruby, and total biomass were quantified using Comstat2. Error bars represent the standard deviation. The data were analyzed using the Student’s *t* test. *p < 0.05.

Given the results from our previous studies showing that *P. gingivalis* generates a biofilm with a protein matrix, we hypothesized that the enhanced biofilm phenotype of *ΔrelΔrshB* strain was due to the accumulation of more matrices. To test this hypothesis, biofilm composition was compared between parent strain and *ΔrelΔrshB* strain by staining with SYPRO Ruby to detect extracellular protein and SYTO 9 to detect bacterial cells. We found that the biomass of *ΔrelΔrshB* biofilms was composed of more cells and more protein matrix (Fig. 6). The ratio of extracellular protein to cells (SYPRO Ruby to SYTO 9) was 1.25 ± 0.11 for the parent strain and 2.76 ± 0.40 for *ΔrelΔrshB* mutant. These results indicate that *ΔrelΔrshB* biofilms contained more extracellular protein per cell. To further examine the mutants for changes in cell surface properties, we performed transmission electron microscopy (TEM) of negatively stained cells. TEM revealed that *ΔrelΔrshB* strain produced an abundance of extracellular substance compared with the parent strain (Supplementary Fig. 3a). The surface of the *ΔrelΔrshB* mutant cells were decorated with negatively stained extracellular substance that extended from the cell surface as fibers to form a meshwork around cells. To investigate the biofilm composition in more detail, we analyzed colony biofilms grown on blood agar plates, using cryo-scanning electron microscopy (Cryo-SEM), as previously described.^41^ Curiously, in our previous study, we discovered that a peptidylarginine deiminase (PPAD) deletion mutant demonstrated a similar enhanced biofilm phenotype, where the biofilm cells were encased in an extensive meshwork of protein matrix. Using the same growth conditions and methodology, the *ΔrelΔrshB* colony biofilms showed a remarkably similar biofilm phenotype (Supplementary Fig. 3b).

**Fig. 7:**
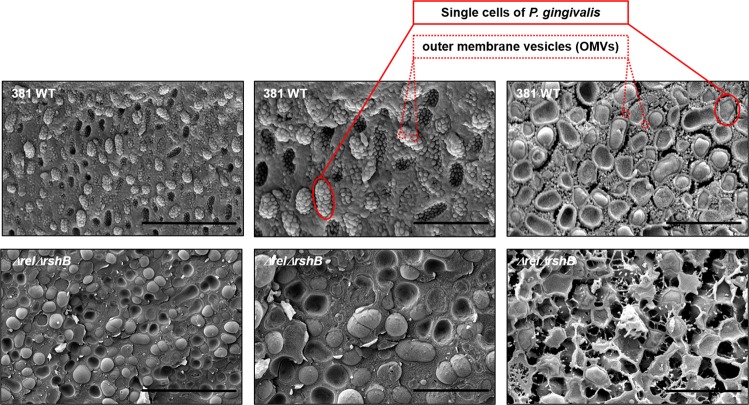
Deletion of the genes encoding RSH proteins in *P. gingivalis* results in increased production of biofilm extracellular matrix. Wild-type strain 381 and *ΔrelΔrshB* colony biofilms grown anaerobically on blood agar plates for 4 days were imaged by Cryo-SEM. The cells of parent strain 381 were surrounded with a large number of outer membrane vesicles (OMVs); however, the cells of the *ΔrelΔrshB* strain were coated in an extracellular matrix that lacked OMVs. The middle panels are magnified versions of the left panels. Scale bar: (top left) 5 µm, (top middle) 2 µm, (top right) 2 µm, (bottom left) 5 µm, (bottom middle) 2 µm, and (bottom right) 2 µm.

With the goal of providing more structural information, we added a fixation step to our cryo-SEM protocol. Using this protocol, the colony biofilms of the parent strain showed easily identifiable bacterial cells covered with a large number of outer membrane vesicles (OMVs) (Fig. 7, top). In contrast, the colony biofilms of the *ΔrelΔrshB* strain showed a smooth surface, and the cells within these biofilms were coated with a matrix-like substance. Importantly, unlike the parent strain, there appear to be very few OMVs produced by the *ΔrelΔrshB* mutant (Fig. 7, bottom). Overall, the data indicate that the enhanced biofilm phenotype of *ΔrelΔrshB* strain is due to accumulation of cell surface and extracellular matrix protein(s).

### Virulence of the ppGpp^0^ mutant is enhanced in the invertebrate *Galleria mellonella* model

**Fig. 8:**
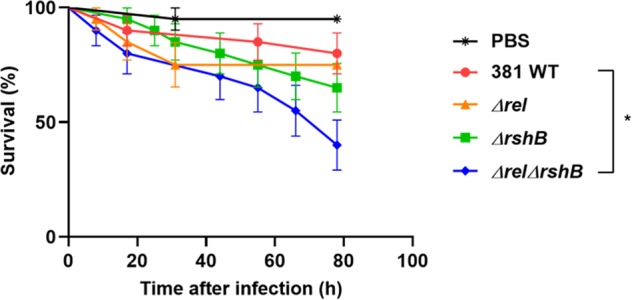
Survival rates of *Galleria mellonella* larvae injected with *P. gingivalis* parent strain 381 or its derivatives. Larvae injected with PBS were used as control. Larvae infected with *ΔrelΔrshB* mutant showed significantly reduced survival rate compared with the parent strain; *p < 0.05 as determined by Log-rank (Mantel–Cox) test. *n* = 10 larvae per treatment. The average of two biological replicates is shown; error bars depict standard deviation.

To investigate the significance of ppGpp in *P. gingivalis* virulence, we used the *G. mellonella* larvae model that possesses an innate immune system. The *Δrel* and *ΔrshB* single mutants killed *G. mellonella* at rates comparable to the parent strain, with similar averages of larvae survival (~75%) after 78 h of infection (Fig. 8). However, as shown in Fig. 8, virulence of the *ΔrelΔrshB* mutant was highly enhanced with ~40% larvae survival after 78 h of infection. Consequently, these findings indicate that ppGpp signaling impacts the virulence of *P. gingivalis* strain 381.

### Gene expression is altered in the *P. gingivalis* ΔrelΔrshB mutant

The gene expression profiles for the parent strain 381 and *ΔrelΔrshB* mutant were analyzed to identify differentially expressed genes that could account for the various *ΔrelΔrshB* mutant phenotypes. As shown in Table 1, a total 94 genes were found to be differentially expressed more than 1.5-fold (*q* value < 0.05) in the *ΔrelΔrshB* mutant compared with the parent strain. Among these genes, 80 genes were upregulated and 14 genes were downregulated. As expected, the *ΔrelΔrshB* mutant demonstrated higher expression levels of the transcripts encoding protein synthesis apparatus, including tRNA and ribosomal proteins, a hallmark of a ppGpp^0^ strain.^42^ In contrast, the expression of a number of genes involved in heme acquisition was significantly downregulated in the *ΔrelΔrshB* mutant compared with the parent strain, specifically HagA (PGN_1733) and PGN_1115, which is a predicted hemagglutinin with high sequence identity (46%) to HagA. To further evaluate the relative transcript levels of genes involved in heme acquisition, we performed quantitative PCR (qPCR) analysis on the gene encoding HagA, hemagglutinin PGN_1115, and the gene encoding HagB (PGN_1904). As shown in Supplemental Fig. 4, the data indicate that all three genes are expressed at lower levels in the mutant. In addition, one of the most prominent changes was the expression of genes involved in T9SS-related proteins. Many genes encoding T9SS cargo proteins (PGN_0654, PGN_0657, PGN_0795, PGN_1115, PGN_1733, and PGN_1767) were downregulated. In contrast, genes encoded T9SS structural proteins, including PorV (PGN_0023), PorT (PGN_0778), PorN (PGN_1673), PorM (PGN_1674), PorL (PGN_1675), PorK (PGN_1676), and PorP (PGN_1677), along with PGN_0274—an extracytoplasmic function (ECF) sigma factor (SigP) that regulates the transcriptional level of T9SS genes, and T9SS C-terminal target domain-containing protein (PGN_0852) was upregulated in the *ΔrelΔrshB* mutant. Notably, the *ΔrelΔrshB* mutant showed significant upregulation of genes involved in cellular metabolism and biosynthetic process. It has been known that (p)ppGpp is important because of its ability to modify global cellular metabolism nearly instantaneously in response to changes in the external environment, thus optimizing growth and promoting survival.^43^ Therefore, since *ΔrelΔrshB* mutant cannot use ppGpp signaling to respond to stress, fitness of *ΔrelΔrshB* mutant may be impaired due to an imbalance between metabolism and protein secretion. Overall, expression of genes involved in heme acquisition, protein synthesis apparatus, cellular metabolism, and T9SS showed significant changes in the *ΔrelΔrshB* mutant compared with the parent strain.

**Table 1:** Differential gene expression of biofilm cells: *ΔrelΔrshB* mutant versus wild type 381.

Annotation	Common name	Predicted product	Fold change^a^ (*ΔrelΔrshB*/WT)
T9SS and its cargo associates/cognate-regulatory genes			
PGN_0023	porV (LptO)	Outer membrane component b-barrel protein, deacylase	3.95
PGN_0295	–	C-terminal domain of Arg- and Lys-gingipain proteinase	0.21
PGN_0300	omp17	OmpH-like protein	1.77
PGN_0654	porQ	T9SS cargo protein	0.29
PGN_0655	–	Hypothetical protein	0.37
PGN_0656	–	Hypothetical protein	0.35
PGN_0657	–	T9SS cargo protein	0.27
PGN_0778	porT	Outer membrane component b-barrel protein	2.28
PGN_0795	–	Fibronectin, hypothetical protein	0.48
PGN_0852	–	T9SS CTD domain and leucine-rich repeats (x7)	2.49
PGN_1115	–	Hemagglutinin	0.47
PGN_1673	porN	Periplasmic component protein	2.52
PGN_1674	porM	Inner membrane component protein	2.36
PGN_1675	porL	Inner membrane component protein	2.12
PGN_1676	porK	Periplasmic component lipoprotein	2.83
PGN_1677	porP	Outer membrane component b-barrel protein	2.35
PGN_1678	–	Hypothetical protein	3.44
PGN_1732	–	Hypothetical protein	0.43
PGN_1733	hagA	Hemagglutinin protein HagA	0.21
PGN_1767	–	T9SS, CTD domain, and IR 46-kDa antigen	0.44
Transcriptional regulation			
PGN_0273	–	Hypothetical protein	4.22
PGN_0274	sigP	RNA polymerase sigma-70 factor ECF subfamily	2.75
PGN_1392	–	Hypothetical protein	1.84
PGN_1393	–	DNA-binding protein HU	1.90
Lipoproteins			
PGN_0154	–	Putative outer membrane lipoprotein	3.43
PGN_0156	–	Putative outer membrane lipoprotein	2.36
PGN_1037	–	Putative lipoprotein	2.91
PGN_1534	–	Hypothetical protein	1.94
PGN_1535	–	Putative lipoprotein	1.89
PGN_1744	–	Putative outer membrane lipoprotein	2.59

Annotation v(33277 ID)	Common name	Predicted product	Fold change^a^ (*ΔrelΔrshB*/WT)
Translational regulation, ribosomal structure, and biogenesis			
PGN_0035	rplS	50S ribosomal protein L19	2.61
PGN_0188	rpmF	50S ribosomal protein L32	2.21
PGN_0279	rplY	50S ribosomal protein L25	2.07
PGN_0394	rpsT	30S ribosomal protein S20	2.39
PGN_0426	rpsC	30S ribosomal protein S3	3.20
PGN_0636	rpmE2	50S ribosomal protein L31	4.02
PGN_0637	htrA	Heat shock-related protease htrA protein	1.93
PGN_0639	rpsF	30S ribosomal protein S6	1.77
PGN_0694	rpmH	50S ribosomal protein L34	2.09
PGN_0963	infC	Translation initiation factor IF-3	2.10
PGN_0965	rplT	50S ribosomal protein L20	2.08
PGN_1580	rpsU	30S ribosomal protein S21	2.04
PGN_1647	rpmA	50S ribosomal protein L27	2.17
PGN_1648	rplU	50S ribosomal protein L21	2.06
PGN_1698	rpsO	30S ribosomal protein S15	2.45
PGN_1703	–	Ribonuclease III	1.98
PGN_1780	–	Putative translation initiation inhibitor, yjgF family	2.03
PGN_1840	rplQ	50S ribosomal protein L17	1.89
PGN_1869	rpsJ	30S ribosomal protein S10	1.86
PGN_1890	rpmG	50S ribosomal protein L33	1.99
PGN_1891	rpmB	50S ribosomal protein L28	2.17
Metabolic and biosynthetic process			
PGN_0024	ispF	2-C-methyl-d-erythritol 2,4-cyclodiphosphate synthase	2.17
PGN_0310	–	Uroporphyrinogen-III synthase	1.90
PGN_0532	–	Magnesium chelatase subunit I	1.88
PGN_0800	–	Electron transfer flavoprotein alpha subunit	1.75
PGN_1239	–	Lipopolysaccharide biosynthesis glycosyltransferase	2.29
PGN_1370	–	NAD-dependent nucleotide–diphosphate–sugar epimerase	2.23
PGN_1375	–	β-ketoacyl–acyl carrier protein reductase	1.94
PGN_1705	acpP	Acyl carrier protein	2.98
Transfer RNA			
PGN_t0009	–	Ile tRNA	2.27
PGN_t0011	–	Glu tRNA	4.03
PGN_t0013	–	Ile tRNA	2.35
PGN_t0029	–	Arg tRNA	2.51
PGN_t0036	–	Ile tRNA	2.26
PGN_t0038	–	Leu tRNA	2.12
PGN_t0049	–	Ala tRNA	2.03

^a^Difference of >1.5-fold (*q* value < 0.05).

## Discussion

The natural habitat for *P. gingivalis* is within the oral microbiome below the gingival margin. This anaerobe not only responds and adapts to many environmental perturbations, such as oxidative stress, but it can also invade host cells and survive within epithelial cells, endothelial cells, and macrophages.^44,45^ Thus, *P. gingivalis* has the ability to adapt to, and persist in, a number of habitats. Here, we have explored the impact of a lack of ppGpp synthesis on *P. gingivalis* physiology and virulence. We have shown that the *rel* and *rshB* genes in *P. gingivalis* encode two RSH proteins that control the levels of ppGpp, and we determined that ppGpp synthesis impacts growth rate, persistence, biofilm formation, and virulence of this strain.

In contrast to the parent strain and the strains with the single mutations, the *ΔrelΔrshB* double mutant showed a number of specific phenotypes not only for the stringent response with regard to growth and survival, but also for the non-stringent processes, including biofilm formation and virulence. Many studies have documented that (p)ppGpp signaling impacts bacterial survival.^46–49^ In this study, only the *ΔrelΔrshB* mutant showed a decrease in survival during the stationary phase, and this altered phenotype was observed in both basal medium with tryptone (CDMTHK) and in rich TSBHK medium. Since the *rel* or *rshB* mutants did not exhibit decreased survival, our results indicate that only basal levels of ppGpp are required for survival of *P. gingivalis* under prolonged culture conditions; yet, without any ppGpp synthesis *P. gingivalis* has difficulty in adapting.

Another interesting finding in this study was that *P. gingivalis* does not appear to accumulate pppGpp. In the previous study by Sen et al., ribosomes were purified from *E. coli* and *P. gingivalis* cells, and the (p)ppGpp levels were investigated in these strains using thin-layer chromatography.^18^ As a positive control, ribosomes isolated from *E. coli* showed large amounts of both ppGpp and pppGpp. Interestingly, ribosomes isolated from *P. gingivalis* accumulated significant amount of ppGpp, but no pppGpp was detected as also observed in our study. The previous study mentioned that it is not clear whether the absence of the pppGpp indicates that this molecule is not synthesized in *P. gingivalis* or that the molecule is unstable. Further investigations are required to determine the reasons for the lack of pppGpp detection in *P. gingivalis*.

Here, we showed that the putative hemagglutinin PGN_1115, HagA, and HagB were expressed at lower levels in the *ΔrelΔrshB* mutant when compared with the parent strain (Supplementary Fig. 4). Furthermore, we showed that deletion of *rel* and *rshB* resulted in less heme binding (Fig. 1a). This result is consistent with a previous study by Connolly et al., which showed that *P. gingivalis* W83 exhibited typical black colonies, whereas *ΔhagBΔhagC* mutant produced white colonies.^3^ In addition, our results showed that the parent strain increased the level of ppGpp in response to hemin starvation (Fig. 1b). Overall, these results support our working hypothesis that ppGpp signaling directly or indirectly regulates the mechanisms involved in heme acquisition in *P. gingivalis*.

**Fig. 9:**
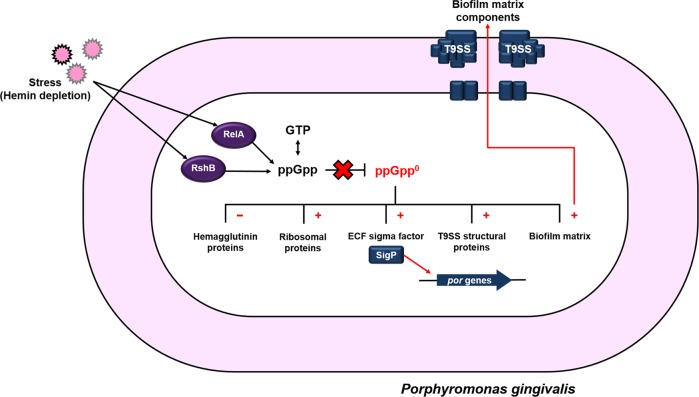
Synthesis of ppGpp impacts global gene expression and biofilm matrix composition. In response to stress such as hemin deprivation, Rel and RshB are responsible for the production of ppGpp. When ppGpp is not synthesized (ppGpp^0^), the expression of genes involved in heme acquisition is downregulated, while there are higher expression levels of genes involved in protein synthesis (tRNA and ribosomal proteins). The ECF sigma factor SigP, which positively regulates T9SS (por genes), is also significantly upregulated in response to a lack of ppGpp. Our working model is that ppGpp synthesis indirectly regulates secretion and localization proteins secreted via T9SS, which in turn impacts outer membrane vesicle production and biofilm matrix formation.

One of the most remarkable discoveries of this study was that ppGpp impacts biofilm matrix composition. Our data show that the *ΔrelΔrshB* mutant forms a biofilm with more total biomass (~2-fold), yet the biofilms consisted of a higher proportion of dead cells. Moreover, the increase in biomass was not only from more cells (dead or alive); in addition, the biofilms consisted of more protein matrix. Furthermore, the matrix composition was also distinct. The matrix of the wild-type biofilm contains copious amounts of OMVs, while the OMV content was negligible in the biofilms formed by the mutant, indicating that ppGpp synthesis affects OMV production. Since transcriptomic analysis discovered that expression of genes encoding the type IX secretion structural proteins was significantly increased in the *ΔrelΔrshB* mutant, it is likely that the enhanced extracellular matrix produced by *ΔrelΔrshB* mutant is made up of proteins secreted by the T9SS (Fig. 9). T9SS is found only in the Bacteroidetes phylum. This novel secretion system plays a role in gliding motility,^50^ and secretion of virulence factors,^51^ and as we recently reported, it is central to *P. gingivalis* surface translocation.^52^ In *P. gingivalis*, T9SS transports select proteins across the outer membrane that are involved in virulence, e.g., gingipains RgpA, RgpB, and Kgp.^9,51^ As shown in Table 1, RNA-seq analysis revealed that an extracytoplasmic function (ECF) sigma factor (PGN_0274) was significantly upregulated in the *ΔrelΔrshB* mutant. ECF sigma factors facilitate changes in gene expression by binding to and guiding the core RNA polymerase. The ECF sigma factor SigP (PGN_0274) has been shown to regulate expression of gingipains, biofilm formation, and the T9SS.^53^ Thus, although many T9SS cargo proteins are downregulated in the ppGpp^0^ mutant, it is possible that the enhanced virulence is due to overproduction of a T9SS-secreted virulence factor. An alternative hypothesis is that dead cells may release internal components, such as peptidoglycan, which for *P. gingivalis* has been shown to be quite toxic.^54^ Additional studies are required to identify the true impact of ppGpp signaling on the T9SS and the pathogenicity of *P. gingivalis*.

This study provides new insights into how ppGpp impacts gene expression in *P. gingivalis*, and increases our understanding of functional links between ppGpp signaling and the virulence of this bacterium. Importantly, our studies suggest that when *P. gingivalis* is intrinsically stressed, i.e., it is unable to synthesize a stress adaptation signal (ppGpp^0^ mutant) or it cannot control the levels of arginine (PPAD mutant), this results in enhanced biofilm formation and a decrease in the production of OMVs. Future studies will determine the significance of this phenotype, but it is intriguing to speculate that being able to inversely regulate protein matrix formation and OMV production may be fundamental to the survival of this bacterium.

## Methods

### Bacterial strains, mutant construction, and complementation

*P. gingivalis* strain 381 (provided by Dr. Howard Kuramitsu, State University of Buffalo, Buffalo, NY) and derivatives were grown on agar plates containing Trypticase Soy Broth (Becton, Dickinson and Company, Franklin Lakes, NJ, USA) supplemented with 5 µg ml^−1^ hemin, 1 µg ml^−1^ menadione, and 5% defibrinated sheep blood (BAPHK) (Northeast Laboratory Services, Winslow, ME, USA) at 37 °C in an anaerobic chamber (Coy Lab Products, Grass Lake, MI, USA) with an atmosphere containing 5% hydrogen, 10% carbon dioxide, and 85% nitrogen. Since the high sequence similarity between *P. gingivalis* strain ATCC 33277 and strain 381, and the nomenclature of genes in strain ATCC 33277 are more detailed than strain 381, we used the names of genes from the strain ATCC 33277 in this report. Mutant strains 381 *Δrel*, 381 *ΔrshB*, and 381 *ΔrelΔrshB* were generated as previously described.^55^ Briefly, primers were designed to generate upstream and downstream products of ~1 kb flanking *rel* or *rshB*, as well as an erythromycin resistance gene (*ermF*) obtained from plasmid pVA2198,^56^ or a tetracycline resistance gene (*tetQ*) obtained from pT-COW.^57,58^ All primers used in this study are listed in Supplementary Table 2. These oligonucleotides were used to prime PCRs using genomic DNA from *P. gingivalis* strain 381 and Phusion high-fidelity PCR master mix with HF buffer according to the manufacturer’s instructions. The products were purified and combined using the NEBuilder HiFi DNA Assembly Master Mix (New England BioLabs, Ipswich, MA, USA) according to the instructions provided by the manufacturer. The final product was mixed with previously frozen cells of *P. gingivalis* and transformed by electroporation.^59^ *P. gingivalis* deletion mutants were maintained by supplementing media with 10 µg ml^−1^ erythromycin or 1 µg ml^−1^ tetracycline. Complementation of the *ΔrelΔrshB* mutant was performed by inserting *rel* (PGN_0465) or *rshB* (PGN_1757) under the control of their native promoter regions into plasmid pC-COW (graciously provided by Ana Duran-Pinedo, University of Florida, Gainesville, FL), generating pC-rel and pC-rshB, respectively. Complemented strains were generated by conjugation as previously described.^60^ In brief, BAPHK containing chloramphenicol (10 µg ml^−1^) was used to select for pC-COW containing *P. gingivalis* strains, and gentamicin (200 µg ml^−1^) was used to counterselect the *E. coli* S17-1 donor. Transconjugants were obtained after 7 days of anaerobic incubation. Clones were isolated, verified by PCR, and maintained on BAPHK containing chloramphenicol (10 µg ml^−1^). To complement the *rel* or *rshB* single-deletion mutants, *rel* or *rshB* were cloned under the control of their native promoter regions into plasmid pT-COW, generating pT-rel and pT-rshB, respectively. Complemented strains were maintained on BAPHK containing tetracycline (1 µg ml^−1^). Details of bacterial strain and plasmid constructions are provided in Supplementary Table 3.

### Bacterial growth

Broth cultures of *P. gingivalis* were grown anaerobically in Tryptic Soy Broth (TSB) medium (Becton, Dickinson and Company, Franklin Lakes, NJ, USA) supplemented with 5 µg ml^−1^ hemin and 1 µg ml^−1^ menadione (TSBHK). Cultures grown overnight in TSBHK were diluted 1:125 in pre-reduced chemically defined medium (CDM) supplemented with 1% tryptone (CDMT), 5 µg ml^−1^ hemin, and 1 µg ml^−1^ menadione (CDMTHK). Bacterial growth was then monitored by measuring the optical density at 600 nm, and presented as the mean ± standard deviations (*n* = 3).

### Detection of (p)ppGpp accumulation patterns

For (p)ppGpp measurements in *P. gingivalis*, overnight cultures grown in TSBHK were diluted 1:125 in pre-reduced CDMTHK. The 0.9-ml cultures of each strain were grown to an optical density at 600 nm of ≈0.5, and prelabeled with 100 μCi of carrier-free [^32^P]orthophosphate (Amersham Biosciences, Piscataway, NJ) aerobically for 4 h at 37 °C. Samples were centrifuged at 13,000 × *g* for 1 min, and 880 μl of the supernatants were discarded. Nucleotide pools were extracted by adding 50 μl of 3 M ice-cold formic acid, followed by two freeze–thaw cycles. Acid extracts were centrifuged briefly, and 30 μl of the supernatant fluids were spotted onto polyethyleneimine (PEI)–cellulose plates (Selecto Scientific, Inc., Suwanee, GA) for separation by thin-layer chromatography (TLC) in 1.25 M KH_2_PO_4_ (pH 3.4). After 30 min of air-drying, the TLC plate was carefully wrapped and exposed to the X-ray film in the radiation cassette for 72 h. The levels of ppGpp were measured using ImageJ software by determining the pixel intensity in each strain.

### Growth and imaging for biofilms

Biofilm assays were performed as previously described with slight modifications.^61^ Briefly, *P. gingivalis* cultures were grown anaerobically in Todd Hewitt Broth (THB) medium (Becton, Dickinson and Company, Franklin Lakes, NJ, USA) supplemented with 5 µg ml^−1^ hemin and 1 µg ml^−1^ menadione (THBHK) for 24 h at 37 °C, subcultured into pre-reduced CDMTHK, and grown to an OD_600_ of ≈1.0, diluted in a pre-reduced CDMTHK to OD_600_ 0.2. Culture aliquots of 200 µl were then placed into uncoated 96-well polystyrene flat-bottom plates under anaerobic conditions at 37 °C for 48 h. Fluorescent imaging was performed in the anaerobic chamber on biofilms grown in 16-well removable coverglass (Grace Bio-Labs, Inc. Bend, Oregon, USA) using SYPRO Ruby Biofilm Matrix Stain (Invitrogen by Thermo Fisher Scientific) and the Invitrogen Live/Dead BacLight Bacterial Viability Kit (Invitrogen by Thermo Fisher Scientific) as per the manufacturer’s instructions, as previously described^41,62^ with slight modification. In brief, culture supernatants (0.2 mL) were removed; wells were washed twice with distilled water; 0.2 mL of mixture (components A [SYTO9] and B [propidium iodide or SYPRO Ruby] were mixed in equal volumes, and 0.6 µl were added per 0.2 ml of distilled water) was added to each well; then the plate was incubated in the dark for 15 min. The dye mixture was removed; wells were washed twice with distilled water; 50 μl of water was added to cover the base of the well. Images of biofilms stained with SYTO9 and propidium iodide, or SYPRO Ruby, were acquired using a Nikon Eclipse Ti inverted fluorescence microscope. SYTO9 fluorescence was detected using the FITC band-pass filter cube. Propidium iodide or SYPRO Ruby fluorescence was detected using the Texas Red bandpass filter cube. Stacked maximum intensity projection images were analyzed using Comstat2 version 2.1.

### Cryo-scanning electron microscopy (Cryo-SEM) of biofilms

Electron microscopy and image analysis were performed by the electron microscopy core of Interdisciplinary Center for Biotechnology Research (ICBR) at the University of Florida. Cryo-SEM experiments were performed using a Quorum PP3010T cryotransfer system (Quorum Technologies, Electron Microscopy Sciences) attached to a Hitachi SU5000FE VP-SEM (Hitachi High Technologies, America). Samples were transported to the UF ICBR EM Core under anaerobic conditions using AnaeroPack (Thermo Scientific) sachets inside Kapak SealPAK Pouches (VWR) and heat-sealed. Colonies were immediately fixed by immersion with 4% formaldehyde and 1% glutaraldehyde in 0.1 M cacodylate buffer, pH 7.24, and kept at 4 °C overnight. Samples were prepared for Cryo-SEM by removal of the colony biofilm from blood agar substrate with a small spatula, and mounted on the edge into the 1-mm slot of a copper stub, with a mixture of colloidal graphite and OCT low-temperature adhesive (Electron Microscopy Sciences). After attaching the sample stub to a transfer shuttle, the colony was vitrified in liquid ethane within a liquid nitrogen reservoir. The ethane-frozen sample was then rapidly plunged into the PrepDek^®^ workstation liquid nitrogen slush at −210 °C under vacuum, and immediately transferred to the Cryo-preparation chamber. The side-mounted colony was fractured, sublimed at −60 °C for 15 min, and sputter coated with platinum for 60 s at 10-mA current in an argon atmosphere. The Cryo-prep chamber returned to −140 °C, at a vacuum of >10−5 mbar, and transferred to the nitrogen gas-cooled cold stage inside the SEM chamber. The sample remained frozen during the imaging at −140 °C, under high vacuum conditions using 5-keV, current emission 194,000 nA, and working distance between 5 and 10 mm.

### *G. mellonella* model of systemic infection

Larvae of *G. mellonella* was used to assess virulence of *P. gingivalis* 381 and its derivatives as described previously.^42^ Briefly, groups of 10 larvae (200–300 mg in weight) were injected with 5 μl of bacterial inoculum containing ~3 × 10^7^ CFU. After injection, larvae were kept at 37 °C, and *G. mellonella* survival was recorded at selected intervals for up to 78 h. The results were analyzed with GraphPad Prism 4.0 software. Experiments were performed independently at least two times with similar results.

### RNA extraction, sequencing, and quantitative PCR (qPCR) analysis

*P. gingivalis* strain 381 and its derivatives were inoculated in TSBHK, and grown for ~24 h. The cultures were subcultured in pre-reduced TSBHK, and grown to an OD_600_ of 1.0. Cultures were diluted 1/5 with fresh pre-reduced TSBHK, and 10-μl aliquots of each culture were spotted on blood agar plates. After 24 h of incubation, cultures were scraped off the plates, and the RNA extraction was performed using the Direct-zol RNA Miniprep kit (Zymo Research), according to the instructions provided by the manufacturer with a slight modification.^63^ RNA samples were delivered to the Gene Expression and Genotyping core of ICBR at the University of Florida. Sample quality determination and sequencing were performed by gene expression and genotyping core in the ICBR.^52^ The program ‘Rockhopper’^64^ was used for aligning sequencing reads to the genome reference of *P. gingivalis* 381. We eliminated any of the sequencing reads with a *q* value > 0.05 and a fold change of <1.5. The qPCR was performed as described previously.^65^ Briefly, cDNA was produced from the same amount of RNA from each sample by using cDNA EcoDry Premix (Clontech). cDNAs were mixed with gene-specific primers and iQ SYBR Green Supermix (Bio-Rad). The qPCR was performed using the CFX96 Real-Time System (Bio-Rad).

### Reporting summary

Further information on research design is available in the Nature Research Reporting Summary linked to this article.

## Supplementary information

Supplemental Figures and Table.

Reporting Summary Checklist

## Data Availability

The authors declare that the data supporting the findings of this study are available within the paper and its Supplementary Information files. Raw sequencing data are available on the NCBI Sequence Read Archive (SRA) under accession number PRJNA600517.
