# Supplementary material for: Synthesis of ppGpp impacts type IX secretion and biofilm matrix formation in Porphyromonas gingivalis
Source: NPJ Biofilms Microbiomes. 2020 Jan 31;6:5. doi: 10.1038/s41522-020-0115-4 (PMC6994654; doi:10.1038/s41522-020-0115-4)
Supplement: Supplementary file 1 — Supplemental Figures and Table. [file 41522_2020_115_MOESM1_ESM.pdf]

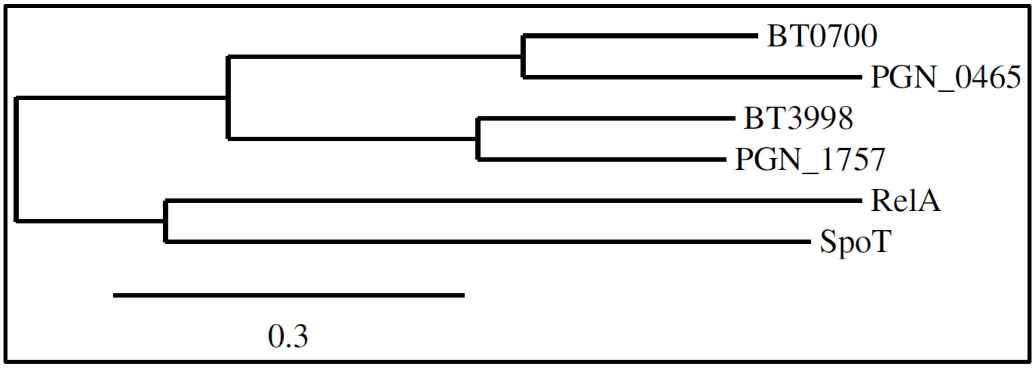

Supplementary Figure 1 Phylogenetic analysis of RSH proteins shows a duplication event for *P. gingivalis* dependent on a Bacteroidetes lineage but independent of Gammaproteobacteria lineage. Protein sequences of RSH enzymes to PGN_0465, PGN_1757 of *P. gingivalis*, *relA*, *spoT* genes of *E. coli*, and *bt0700*, *bt3998* genes of *Bacteroides thetaiotaomicron* were obtained from the database resources of the National Center for Biotechnology Information. Phylogenetic analyses were conducted using MABL Phylogeny.fr "One Click" mode (http://www.phylogeny.fr/). Branch lengths represented by a scale bar indicate genetic change.

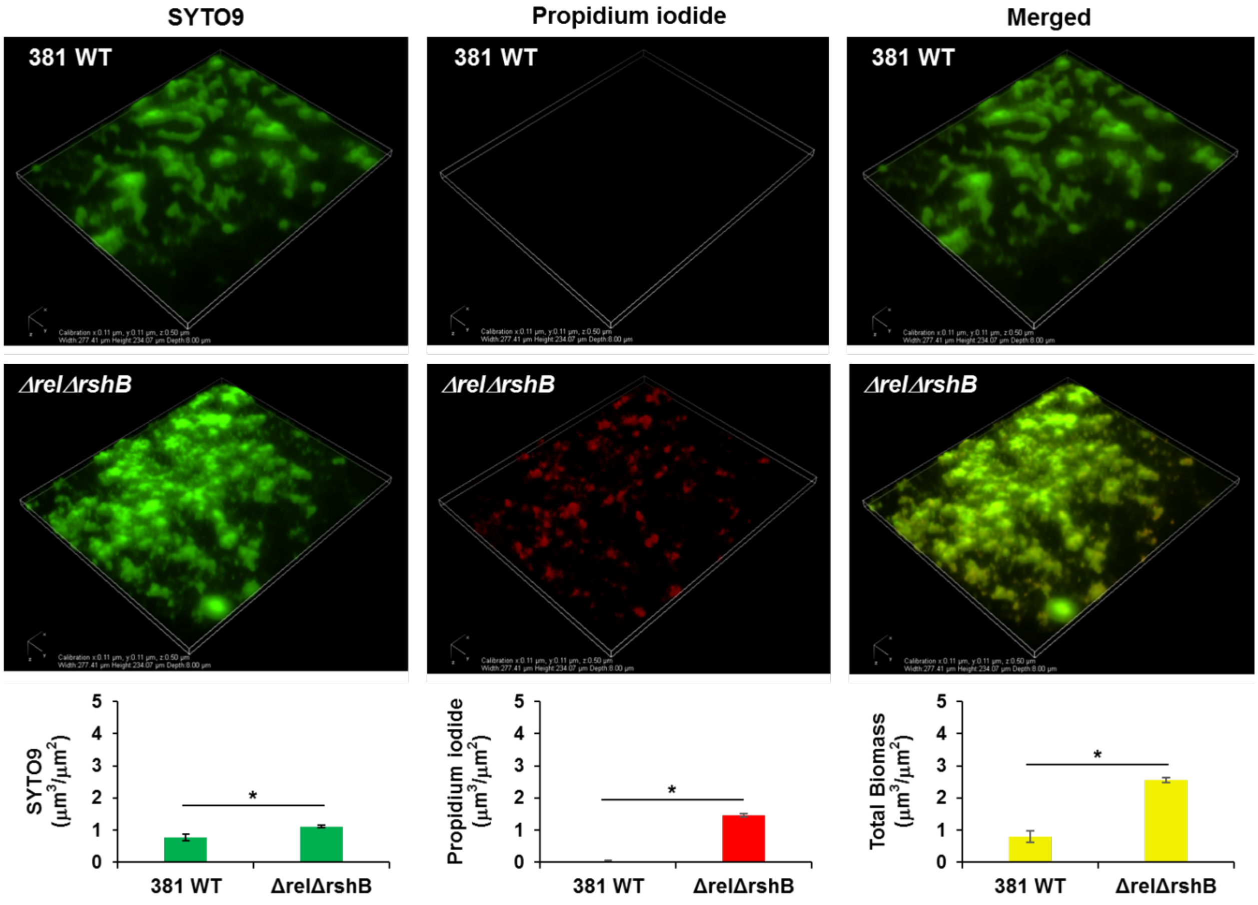

Supplementary Figure 2 Live/Dead cell viability assays for the *P. gingivalis* biofilms. Wild-type 381 and *ΔrelΔrshB* mutant were grown for 48 h on glass, stained with SYTO 9 (Green) and propidium iodide (PI; red) to indicate total and dead cell populations, respectively. A z-stack (17 images per stack) was collected using Nikon imaging software (NIS-elements AR 4. 30. 02 64-bit). A graticule size: 50.00 µm (Width: 277.41 µm, Height: 234.07 µm, Depth: 8.00 µm). The images shown are representative of three independent experiments. SYTO 9, propidium iodide, and total biomass were quantified using Comstat2. Error bars represent the standard deviation. The data were analyzed using the Student's *t*-test. *$p < 0.05$

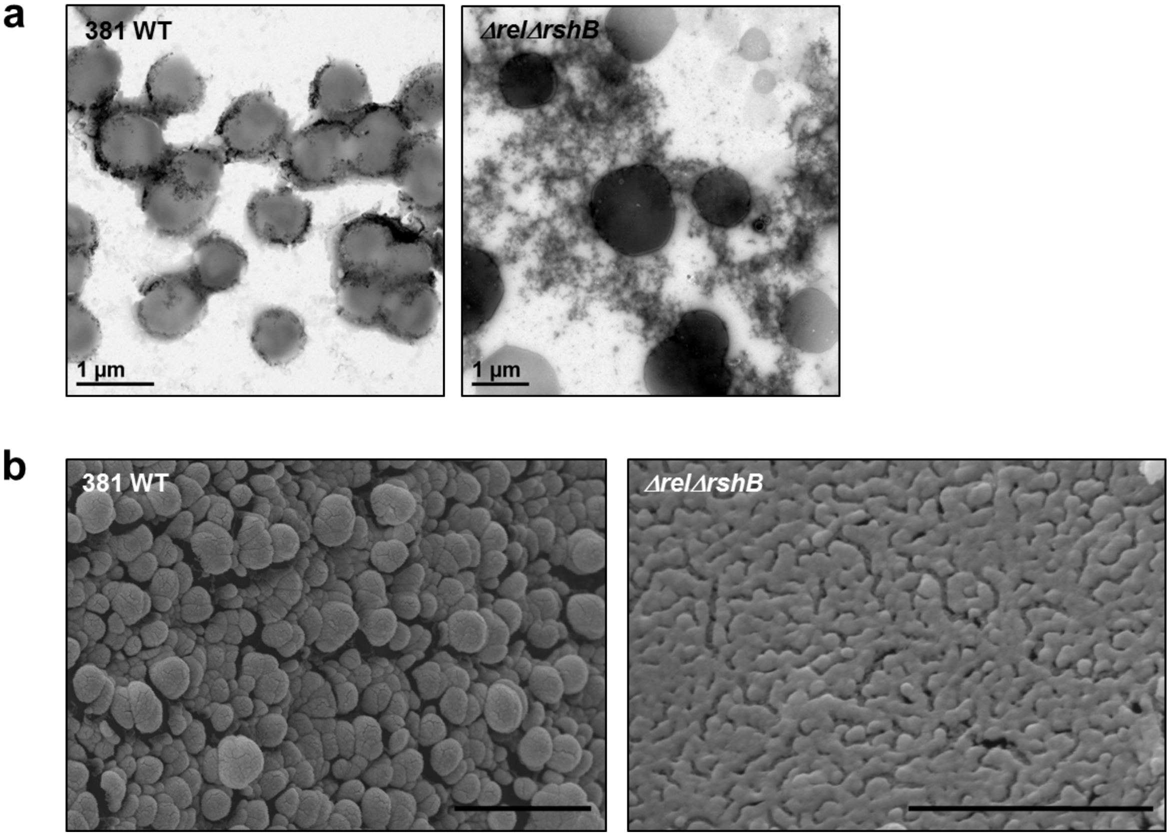

Supplementary Figure 3 Deletion of the genes encoding RSH proteins in *P. gingivalis* results in increased matrix production. **a** Transmission electron microscopy (TEM) revealed that *ΔrelΔrshB* mutant produced an abundance of extracellular matrix more than the parent strain 381. Bacterial cells grown anaerobically on blood agar plates for 4 days were negatively stained with 0.5% aqueous uranyl acetate and then imaged by TEM as previously described.[1] Scale bar: (left) 1.0 µm and (right) 1.0 µm. **b** Parent strain 381 and *ΔrelΔrshB* colony biofilms grown anaerobically on blood agar plates for 4 days were imaged by Cryo-SEM as previously described.[1] Using the same growth conditions and methodology as our previous study, the *ΔrelΔrshB* colony biofilms showed a remarkably similar altered biofilm matrix as the peptidylarginine deiminase (PPAD) deletion mutant. Scale bar: (left) 1.0 µm and (right) 1.0 µm.

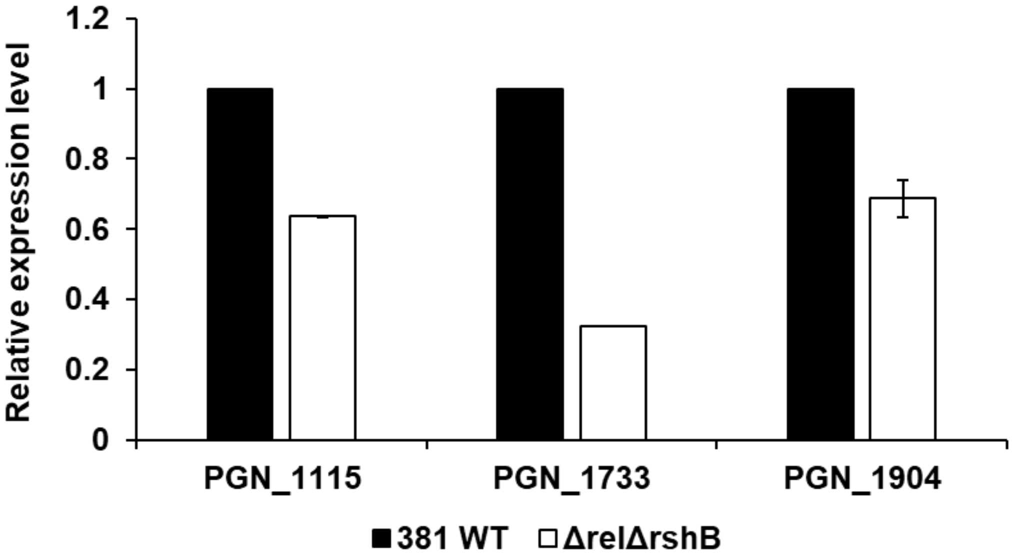

	PGN_1115	PGN_1733	PGN_1904
381 WT	1	1	1
ΔrelΔrshB	0.64 ± 0.0003	0.33 ± 0.0003	0.69 ± 0.05

Supplementary Figure 4 Quantitative PCR analysis of genes involved in hemagglutination in *P. gingivalis*. The relative transcript levels of genes involved in hemagglutination, including hemagglutinin (PGN_1115), HagA (PGN_1733), and HagB (PGN_1904) were determined on the same RNA samples used for data presented in Table 1. The qRT-PCR was performed as described previously.[2,3] The results are presented as the relative levels (mean ± S.D. of triplicate determinations) compared to the transcript levels of the parent strain 381. Error bars represent standard deviations of triplicate replicates.

Supplementary Table 1. Sequence alignment of *P. gingivalis* genes (Pg_Rel, Pg_RshB) with (p)ppGpp synthetase/hydrolase from Gammaproteobacterium *Streptococcus equisimilis* (RelSeq) and Bacteroides genes homologous (BT_3998, BT_0700).

Gene	RelSeq Hydrolysis domain summary<br>Key residues changed (out of 29)	RelSeq Synthesis domain summary<br>Key residues changed (out of 18)
**Pg_RshB**	**6**	**8**
**BT_3998**	**7**	**8**
**Pg_Rel**	**22**	**5**
**BT_0700**	**19**	**5**

```
RelSeq      --------------------MAKEINLTGEEVVALAAKYMN---ETDAAFVKKALDYATAA   38
Pg_RshB     MEQDASAKNPDIETENKGAISEEEALIQREYSLLIQDYLNSNHRRKVEKIDKAFHLAKDA   60
BT_3998     -----------MDNITPK-EIADEEMINQAFQELLNDYLHTKHRKRVEIITKAFNFANQA   48
Pg_Rel      -------MTIHDNT---LFSADERSRFLSSYASLIRTLAPDRQ--LVKKVRSLLSRFTEL   48
BT_0700     -----------MDD---FFTSEEKKELFSLYRHLLQSAGDTIFWRDCQKLKKHLIKAAQC   46

RelSeq      HFYQVRKSGEPYIVHPIQVAGILA-DLHLDAVTVACGFLHDVVEDTDITLDNIEFDFGKD   97
Pg_RshB     HAGAKRRSGEPYILHPIAVARIVCQEIGLGSTSICCALLHDVVEDTEYTVEDMRDMFGDK   120
BT_3998     HKGIKRRSGEPYIMHPIAVAQIVCNEIGLGSTSICAALLHDVVEDTDYTVEDIENIFGPK   108
Pg_Rel      GCFDRDKNGMHGLLRNMEVARIASCEIGLGIETIGALLFYRPAMKGLMRPEEIAELMGED   108
BT_0700     NGLQRNNFGMNPVIRDLQTAVIVAEEIGMKGSCLVGIMLHEIVKAHILSIEEVNAEYGED   106

RelSeq      VRDIVDGVTKLGKVEYKSHEEQLAENHRKMLMAMSKDIRVILVKLADRLHNMRTLKHLRK   157
Pg_RshB     VAQIVDGLTKISGEVFSN-SSAQAENFRKLILTMNDDIRVILIKIADRLHNMRTLDSMLP   179
BT_3998     IAQIVDGLTKISGGIFGDRASAQAENFKKLLLTMSNDIRVILIKIADRLHNMRTLGSMLP   168
Pg_Rel      VARLIDLLLKTS-EIYMRNTAINTANFEHFLLSIAEDIRVVLLIIADRSYLLRQAKDMVC   167
BT_0700     VGSIIKGLVKTN-ELYSKSPAIESENFRNLLLSFAEDMRVILIMIADRVNIMRQIKDTGN   165

RelSeq      D-KQERISRETMEIYAPLAHRLGISRIKWELEDLAFRYLNETEFYKISHMMNEKRREREA   216
Pg_RshB     A-KQMKIAGETQYVYAPLAHRLGLFAIKSELEDLSFKYEHPQEYEAIKEKIRITEQHRLE   238
BT_3998     N-KQYKIAGETLYIYAPLANRLGLYKIKTELENLSFKYEHPEEYAEIEEKLNATAAERDK   227
Pg_Rel      AEERTALAAEVSYLYAPLAHRLGLYAIKSEMEDLCLKYTDRQTFDFIKRKLGETKRSRDA   227
BT_0700     EEDRVKVANEAAYLYAPLAHKLGLYKLKSELEDLSLKYTQRETYYFIKEKLNETKASRDK   225

RelSeq      LVDDIVTKIKSYTTEQ--GLFGDVYGRPKHIYSIYRKMRDKKKRFDQIFDLIAIRCVMET   274
Pg_RshB     MFEHFAAPLKQRFAGM--DMSYEMKARVKSVYSIWKKMEKKGVPFEEVYDLFAVRIIFES   296
BT_3998     VFNDFTAPIRTQLDKM--GLKYRILARVKSIYSIWNKMQTKHVPFEEIYDLLAVRIIFEP   285
Pg_Rel      YIEAFIAPLRRRLDEALPNIPYEMKGRTKSINSIRNKLRQQGIEFESIYDLFAIRIILDV   287
BT_0700     YIASFIDPIQKKVKEA--GLKFDIKGRTKSIHSIWNKIQKQKTPFEGIYDLFAIRIILDS   283

RelSeq      QS-------DVYAMVGYIHELWRPMPGRFKDYIAAPKANGYQSIHTTVYGPKGP-IEIQI   326
Pg_RshB     SPT-IPDKNRCWEIYSAITDIYRNRPDRIRDWVSNPKSNGYQALHLTVMGPDGEWVEVQI   355
BT_3998     RNV-EEELNDCFDIYVSISKIYKPHPDRLRDWVSHPKANGYQALHVTLMGNNGQWIEVQI   344
Pg_Rel      PV--KDEKAACWHVYSIITDMYQPNPQRMKDWISIPKSNGYESLHVTVMGPQNRWVEVQI   345
BT_0700     EPDPAKEKQECWQVYSIVTDMYQPNPKRLRDWLSIPKSNGYESLHITVMGPEGRWVEVQI   343

RelSeq      RTKEMHQVAEYGVAAHWAYKKGVRGKVNQAEQKVGMNWIKELVEL---QDASNGDAVDFV   383
Pg_RshB     RSRRMDDIAEKGFAAHWKYKG-DNVEED----SELEKWLSTIQEILENPN---PNALDFL   407
BT_3998     RSERMNDVAEQGFAAHWKYKEGGGSEDE----GELEKWLKTIKEILDDPQ---PDAIDFL   397
Pg_Rel      RSRRMDEVAERGLAAHWKYKG---IKSE----SGLDEFLTSVRETLEARDHSSDDSAETV   398
BT_0700     RTRRMDEIAERGLAAHWRYKG---IKGE----TGLDEWLTSVREALENAD---NDSLKVM   393
```

Supplementary Table 2. Primers used in this study

Name	Sequence (5'-3')	Purpose
PGN0465_1100bpUP_FW	CCGCCACCATGTACATATTCAG	Sequencing of *Δrel*::Erm
PGN0465_1100bpDW_RV	TACGATCCCCTACCTTTTCGCTG	Sequencing of *Δrel*::Erm
pUC19_FW	GCGGGCCTCTTCGCTATTAC	Sequencing of *Δrel*::Erm on plasmid
pUC19_RV	GATGCGGTATTTTCTCCTTAC	Sequencing of *Δrel*::Erm on plasmid
relA_up_FW	ATGCGTAAGGAGAAAATACCGCATCGTGAAGGTGAGGGTCATATTC	Deletion of *Δrel*::Erm or *Δrel*::Tet
relA_up_RV	CGGAAGCTATCGGGAGGGATACAGGATGGGATAATG	Deletion of *Δrel*::Erm
Pro_ermF_FW	TCCTGTATCCCTCCCGATAGCTTCCGCTATTG	Deletion of *Δrel*::Erm
Pro_ermF_RV	TTGCAAGGGGCTGTCATCTTGACAACCACCC	Deletion of *Δrel*::Erm
relA_down_FW	GTTGTCAAGATGACAGCCCCTTGCAAGTCTTTAG	Deletion of *Δrel*::Erm
relA_down_RV	CTGGCGTAATAGCGAAGAGGCCCGCTGAGGTCGCTGTCTCATG	Deletion of *Δrel*::Erm or *Δrel*::Tet
PGN1757_1100bpUP_FW	CTATCCGCTGAAGATTGCCGAC	Sequencing of *ΔrshB*::Erm
PGN1757_1100bpUP_RV	CGTTGGCTACGGATATGACTGC	Sequencing of *ΔrshB*::Erm
pUC19_HindIII	AAGCTTGGCGTAATCATGG	Sequencing of *ΔrshB*::Erm on plasmid
pUC19_EcoRI	GAATTCACTGGCCGTCGTTTTACAACG	Sequencing of *ΔrshB*::Erm on plasmid
spoT_UP_FW	TTGTAAAACGACGGCCAGTGAATTCAAAGATGATACAAAAGGCATTC	Deletion of *ΔrshB*::Erm

spoT_UP_RV	CGGAAGCTATCGGAATCTATTCTCA ATCTACAATGTG	Deletion of *ΔrshB*::Erm
spoT_proermF_FW	TTGAGAATAGATTCCGATAGCTTCC GCTATTG	Deletion of *ΔrshB*::Erm
spoT_proermF_RV	AGCCTCTTGGCCATCATCTTGACAA CCACCC	Deletion of *ΔrshB*::Erm
spoT_DW_FW	GTTGTCAAGATGATGGCCAAGAGGC TCTCCC	Deletion of *ΔrshB*::Erm
spoT_DW_RV	CTATGACCATGATTACGCCAAGCTT ACGATGATCAGTTCGCTTGTTCAC	Deletion of *ΔrshB*::Erm
up flnk of relA_R	attcgttgtttatGAGGGATACAGGATGGGA TAATG	Deletion of *Δrel*::Tet
tetQ (w/P) for relA_F	ttgcaaggggctgTTATTTTGATGACATTG ATTTTTGG	Deletion of *Δrel*::Tet
tetQ (w/P) for relA_R	tcctgtatccctcATAAACAACGAATTATCT CCTTAAC	Deletion of *Δrel*::Tet
down flnk of relA_F	gtcatcaaaataaCAGCCCCTTGCAAGTCTT TAG	Deletion of *Δrel*::Tet
qRT_PGN_1115_FW	CGTGGTTGGCAAGACCATTG	qRT-PCR of PGN_1115
qRT_PGN_1115_RV	TACCTAAGCGTATCGCGTCC	qRT-PCR of PGN_1115
qRT_PGN_1733_FW	ACAGAACCTGACGGCAGAAC	qRT-PCR of PGN_1733
qRT_PGN_1733_RV	GAAGTCTTCGTTCAGAACTTCCGC	qRT-PCR of PGN_1733
qRT_PGN_1904_FW	GAGAAGCAGCCGTGGAGAAG	qRT-PCR of PGN_1904 and PGN_1906
qRT_PGN_1904_RV	CCGTTCTCGGCTACGAATGT	qRT-PCR of PGN_1904 and PGN_1906
proPGN0465_FW	acgcagtcaggcaccgtgtATGGATAGTCAGG TGGCTTC	PGN_0465 cloning in pT-COW or pC-COW
proPGN0465_RV	aggtgccgccggcttccattTCAATGTCGTTCG GCCGAC	PGN_0465 cloning in pT-COW or pC-COW
proPGN1757_FW	acgcagtcaggcaccgtgtACGAGCAAGCCAT CCAGCG	PGN_1757 cloning in pT-COW or pC-COW
proPGN1757_RV	aggtgccgccggcttccattTTATGATATGCGT CCGACAGACTG	PGN_ cloning in pT-COW or pC-COW

Supplementary Table 3. Strains and plasmids used in this study

**Strain (relevant genotype)**	**Source or reference**
P. gingivalis **strains**	
381 (wild-type)	H. Kuramitsu, State University of Buffalo, Buffalo, NY
Δrel::Erm (Em^r) in strain 381	This study
ΔrshB::Erm (Em^r) in strain 381	This study
Δrel::Tet (Tc^r); *ΔrshB*::Erm (Em^r) in strain 381	This study
381/pT-COW	This study
Δrel/pT-COW	This study
Δrel/pT-rel	This study
ΔrshB/pT-COW	This study
ΔrshB/pT-rshB	This study
381/pC-COW	This study
ΔrelΔrshB/pC-COW	This study
ΔrelΔrshB/pC-rel	This study
ΔrelΔrshB/pC-rshB	This study
E. coli **strain**	
NEB 5α	NEB
S17-1	[4]
**Plasmids**	
pUC19	NEB
pT-COW (Amp^R and Tc^R in *E. coli*; Tc^R in *P. gingivalis;* Mob^+ Rep^+)	[5]
pT-rel (Amp^R in *E. coli*; Tc^R in *P. gingivalis;* Mob^+ Rep^+)	This study
pT-rshB (Amp^R in *E. coli*; Tc^R in *P. gingivalis;* Mob^+ Rep^+)	This study
pC-COW (Amp^R and Tc^R in *E. coli*; Cm^R in *P. gingivalis;* Mob^+ Rep^+)	Ana Duran-Pinedo, University of Florida, Gainesville, FL

pC-rel (Amp^R in *E. coli*; Cm^R in *P. gingivalis;* Mob^+ Rep^+)	This study
pC-rshB (Amp^R in *E. coli*; Cm^R in *P. gingivalis;* Mob^+ Rep^+)	This study

Supplementary References

1 Vermilyea, D. M., Ottenberg, G. K. & Davey, M. E. Citrullination mediated by PPAD constrains biofilm formation in *P. gingivalis* strain 381. *NPJ Biofilms Microbiomes* **5**, 7, doi:10.1038/s41522-019-0081-x (2019).

2 Kim, H. M., Park, Y. H., Yoon, C. K. & Seok, Y. J. Histidine phosphocarrier protein regulates pyruvate kinase A activity in response to glucose in *Vibrio vulnificus*. *Mol Microbiol* **96**, 293-305, doi:10.1111/mmi.12936 (2015).

3 Park, Y. H., Lee, C. R., Choe, M. & Seok, Y. J. HPr antagonizes the anti-sigma70 activity of Rsd in *Escherichia coli*. *Proc Natl Acad Sci U S A* **110**, 21142-21147, doi:10.1073/pnas.1316629111 (2013).

4 Matsumoto-Mashimo, C., Guerout, A. M. & Mazel, D. A new family of conditional replicating plasmids and their cognate *Escherichia coli* host strains. *Res Microbiol* **155**, 455-461, doi:10.1016/j.resmic.2004.03.001 (2004).

5 Gardner, R. G., Russell, J. B., Wilson, D. B., Wang, G. R. & Shoemaker, N. B. Use of a modified *Bacteroides*-*Prevotella* shuttle vector to transfer a reconstructed beta-1,4-D-endoglucanase gene into *Bacteroides uniformis* and *Prevotella ruminicola* $B_1$4. *Appl Environ Microb* **62**, 196-202 (1996).
